# Association of multiple inflammatory index markers with overactive bladder syndrome: A cross-sectional study results from the NHANES 2005 to 2018

**DOI:** 10.1097/MD.0000000000045154

**Published:** 2025-10-10

**Authors:** Mingchu Jin, Jie Xu, Heng Liu, Yu Zhou, Haidong Hao, Yutang Yuan, Hongtao Jia

**Affiliations:** a Department of Urology, Renmin Hospital, Hubei University of Medicine, Shiyan, Hubei Province, PR China.

**Keywords:** inflammatory index markers, NHANES, overactive bladder, Systemic Inflammatory Index, Systemic Inflammatory Syndrome Index, The Systemic Inflammatory Response Index

## Abstract

The Systemic Inflammatory Response Index (SIRI), neutrophil/lymphocyte ratio, Aggregate Inflammatory Syndrome Index, monocyte/lymphocyte ratio, platelet/lymphocyte ratio, and Systemic Inflammatory Index are readily accessible inflammatory biomarkers. The pathogenesis of overactive bladder (OAB) is considered related to the inflammatory response. This study aims to explore the relationships between these biomarkers and OAB and to assess their potential as predictors of disease onset. This study is a retrospective cross-sectional analysis utilizing data from the National Health and Nutrition Examination Survey from 2005 to 2018. Given the preexisting nature of the National Health and Nutrition Examination Survey data collection, this study retrospectively evaluates the association between systemic inflammatory markers and OAB based on previously recorded clinical and demographic variables. A total of 32,115 participants were analyzed. Our findings demonstrated a significant association between systemic inflammatory markers and OAB. Among the inflammatory indices, SIRI (odds ratio [OR] = 1.184, 95% confidence interval [95% CI]: 1.150–1.220, *P* < .001), neutrophil/lymphocyte ratio (OR = 1.116, 95% CI: 1.091–1.141, *P* < .001), and monocyte/lymphocyte ratio (OR = 2.632, 95% CI: 2.116–3.275, *P* < .001) exhibited the strongest association with OAB. Receiver operating characteristic analysis indicated that SIRI had the highest predictive value (area under the curve = 0.549, 95% CI: 0.541–0.557, *P* < .05). These findings suggest that systemic inflammation may play a crucial role in OAB pathogenesis. Levels of the biomarkers were significantly correlated with the incidence of OAB. In the U.S., individuals with elevated levels of these markers exhibited a higher prevalence of OAB.

## 1. Introduction

Urinary urgency, either with or without urge incontinence, is the hallmark of overactive bladder syndrome (OAB).^[1]^ Typically, OAB is accompanied by increased nocturia and urine frequency.^[2,3]^ The recognition of OAB primarily relies on patient-reported symptoms, with diagnosis currently based on patient history,^[4]^ volumetric examination and urinalysis. Standardized survey measures have a major role in determining the intensity of symptoms and their influence on quality of life. The prevalence of OAB varies across different countries and regions, potentially due to factors such as race and lifestyle habits. OAB can significantly impact a patient’s quality of life and social functioning.^[5]^ Studies indicate a high prevalence of OAB in the U.S. population, affecting up to 16% of men and 16.9% of women, with substantial annual healthcare costs associated with its treatment.^[6,7]^ Several research works have proposed a connection between different immune-inflammatory reactions and the incidence of OAB, although the underlying mechanisms remain unclear.^[8,9]^ According to several studies, OAB patients have considerably greater serum and urine levels of prostaglandins, C-reactive protein and nerve growth factor than non-OAB patients.^[10,11]^

Inflammation may contribute to symptoms such as frequent urination and urinary urgency, suggesting that it might have played a role in the emergence of OAB.^[12,13]^ Various systemic inflammation indicators, including the Systemic Inflammatory Response Index (SIRI), neutrophil/lymphocyte ratio (NLR), Aggregate Inflammatory Syndrome Index (AISI), platelet/lymphocyte ratio (PLR), monocyte/lymphocyte ratio (MLR), Systemic Inflammatory Index (SII), have been utilized to predict a range of inflammation-related diseases.^[14,15]^ Recent studies suggest that systemic immune-inflammatory markers, including the SII, play a role in the pathogenesis of OAB. SII, which integrates platelet, neutrophil, and lymphocyte counts, has been linked to inflammatory and immune processes that contribute to bladder dysfunction. Previous research has demonstrated that elevated SII levels are associated with increased OAB severity, suggesting that systemic inflammation may be a key factor in disease onset and progression.This revision provides a concise summary of prior research, emphasizing the relevance of SII in OAB pathophysiology, as requested by the reviewer.^[16]^ Thus, the goal of this research was to determine whether the onset of OAB is directly correlated with the levels of SIRI, NLR, AISI, PLR, MLR, and SII in participants of the National Health and Nutrition Examination Survey (NHANES). OAB is a prevalent condition affecting both men and women, significantly impairing quality of life and leading to substantial healthcare costs. While OAB is primarily diagnosed based on symptomatology, growing evidence suggests that systemic inflammation plays a crucial role in its pathophysiology. Various systemic inflammatory indices, including the SII, SIRI, and NLR, have been linked to chronic inflammatory diseases. Recent studies indicate that elevated levels of these biomarkers may correlate with OAB severity, yet their predictive utility remains underexplored. This study aims to investigate the association between multiple inflammatory markers and OAB using a large-scale retrospective analysis of NHANES data (2005–2018), providing new insights into their diagnostic potential.

## 2. Materials and methods

### 2.1. Study population

**Figure 1.**
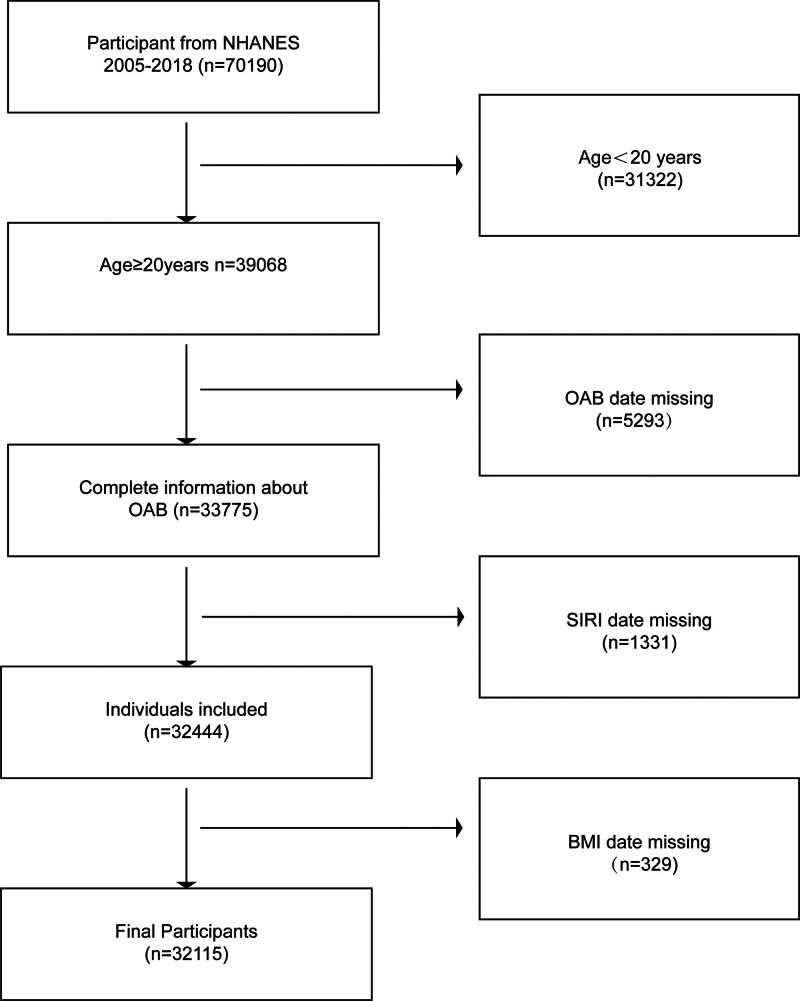
Flowchart of participant selection. NHANES = National Health and Nutrition Examination Survey.

Data were taken from the NHANES from 2005 to 2018. We ruled out the participants under 20 years of age (n = 32,322), those whose data are missing on OAB (n = 5293), those whose data are missing on inflammatory markers (n = 1331), and those with missing body mass index (BMI) data (n = 329), resulting in a final sample of 32,115 eligible individuals (Fig. 1). Ultimately, the study included 24,198 non-OAB participants and 7917 OAB patients. The age range of participants was 20 to 80 years.

### 2.2. Survey description

In order to guarantee that the estimated numbers are representative of the civilian, noninstitutionalized population in the United States, NHANES used a sophisticated sampling method that incorporates oversampling. The National Center for Health Statistics provides recommendations on creating acceptable multiyear weights by dividing the 2-year weights by the number of cycles collected, as providing weights for every possible combination across multiple cycles is not feasible. Following this procedure, we derived the necessary weights to combine the 2005 to 2018 NHANES 2-year units for our analysis of potential associations between OAB and various systemic inflammatory indices during this extended period.

### 2.3. Data acquisition

Multiple inflammation markers were obtained from laboratory data, with a complete blood count performed using an automated hematology analyzer. The systemic inflammation indices were calculated as follows: SIRI = neutrophil × monocyte/lymphocyte; NLR = neutrophil/lymphocyte; AISI = platelet × neutrophil × monocyte/lymphocyte; PLR = platelet/lymphocyte; MLR = monocyte/lymphocyte; SII = platelet × neutrophil/lymphocyte.

### 2.4. Selection of covariates

The following covariates were included in the analysis: age, sex, race/ethnicity, education level, marital status, household income (poverty income ratio), smoking status, BMI, and comorbidities such as hypertension and diabetes. BMI was calculated using measured weight (kg) divided by height squared (m²). Participants were classified into 3 BMI categories: normal weight (BMI < 25.0 kg/m²), overweight (25.0–29.9 kg/m²), and obese (≥30.0 kg/m²). The presence of hypertension and diabetes was determined based on self-reported physician diagnoses and laboratory measurements, consistent with NHANES classification criteria.

OAB history was obtained through a face-to-face interview conducted by trained staff using a standardized questionnaire. The intensity and existence of urge incontinence were determined by 2 questions^[17]^: “During the past 12 months, have you (or has SP) leaked or lost control of even a small amount of urine with an urge to urinate and couldn’t get to the toilet fast enough?” and “How frequently does this occur?” Nocturia was assessed by asking, “During the past 30 days, how many times per night did you (or SP) typically get up to urinate, from the time you (or SP) went to bed at night until getting up in the morning?” An overall score for symptoms of overactive bladder syndrome ≥ 3 was used to diagnose OAB (Table 1).^[17]^ These measures are consistent with previous studies utilizing the NHANES database.

**Table 1:** Criteria for conversion of symptom frequencies recorded in NHANES and OABSS scores.

According to NHANES score	According to OABSS score
Urge urinary incontinence frequency	Urge urinary incontinence score
Never	0
Less than once a month	1
A few times a month	1
A few times a week	2
Every day or night	3
Nocturia frequency	Nocturia score
0	0
1	1
2	2
3	3
4	3
5 or more	3
When total score ≥ 3, the diagnosis is OAB	

NHANES = National Health and Nutrition Examination Survey, OAB = overactive bladder, OABSS = Overactive Bladder Symptom Score.

### 2.5. Statistical analysis

The Centers for Disease Control and Prevention standards were followed in all statistical analyses, which used the proper NHANES weights to take into consideration the intricate multistage cluster sampling design. Categorical data are shown as proportions, and continuous variables are provided as weighted means with standard errors. Weighted Student *t* tests for continuous variables and weighted *χ*² tests for categorical variables were used to evaluate differences between groups. Additionally, several indicators of systemic inflammation were divided into quartiles, with the lowest quartile serving as the reference. Age-standardized prevalence estimates and 95% confidence intervals were calculated for each level of systemic inflammation marker. Survey-weighted multivariate logistic regression was used to model independent associations between systemic inflammatory markers and the possibility of overactive bladder, adjusting for potential demographic confounders, hypertension, diabetes, and BMI. Stratified analyses by age, sex, race, marital status, education level, and poverty rate were conducted to examine subgroup susceptibility to population-related differences. Statistical tests were 2-sided, with significance set at *P* < .05. R version 4.2 and Free Statistics version 1.8 were used for all of the analyses.

## 3. Results

### 3.1. Participant characteristics

Table 2 presents the results comparing SIRI, NLR, AISI, PLR, MLR, SII levels, and important demographic factors between patients with OAB and those without OAB. The table shows that, out of all the participants (n = 32,115), compared to the non-OAB group, the mean levels of SIRI, NLR, AISI, PLR, MLR, and SII were considerably greater in the OAB group (*P* < .001). A comparable noteworthy distinction was observed in mean age, with OAB patients having a mean age of 59.18 years compared to 47.31 years for those without OAB. Additionally, the proportion of females was higher in the OAB group (63.7% vs 46.8% in the non-OAB group, *P* < .001). Racial and ethnic breakdowns revealed that the majority of participants were White or Black. Marital status, education level, poverty ratio, and the frequency of coexisting conditions (including hypertension, diabetes, and obesity) were all significantly associated with the presence of OAB.

**Table 2:** Baseline characteristics of NHANES participants between 2005 to 2018 (n = 32115).*

	Total	OAB		*P* value
		No (24,198)	Yes (7917)	
*Gender*				<.00001
Male	15,747 (49.033%)	12,873 (53.199%)	2874 (36.302%)	
Female	16,368 (50.967%)	11,325 (46.801%)	5043 (63.698%)	
Age	50.242 ± 17.516	47.315 ± 16.967	59.186 ± 16.081	<.00001
*Race*				<.00001
Mexican American	5065 (15.771%)	3882 (16.043%)	1183 (14.943%)	
Other Hispanic	3081 (9.594%)	2340 (9.670%)	741 (9.360%)	
Non-Hispanic White	13,957 (43.459%)	10,459 (43.223%)	3498 (44.183%)	
Non-Hispanic Black	6646 (20.694%)	4688 (19.374%)	1958 (24.732%)	
Other race	3366 (10.481%)	2829 (11.691%)	537 (6.783%)	
*Education*				<.00001
Less than high school	7808 (24.333%)	5291 (21.881%)	2517 (31.833%)	
High school and more than high school	24,280 (75.667%)	18,890 (78.119%)	5390 (68.167%)	
*Marital*				<.00001
Widowed/divorced/separated	7235 (22.539%)	4596 (19.002%)	2639 (33.350%)	
Married/living with partner	19,518 (60.804%)	15,171 (62.724%)	4347 (54.935%)	
Never married	5347 (16.657%)	4420 (18.274%)	927 (11.715%)	
*PIR*				<.00001
0–1.5	8976 (27.986%)	6351 (26.285%)	2625 (33.182%)	
1.5–3.5	11,232 (35.020%)	8369 (34.637%)	2863 (36.190%)	
>3.5	11,865 (36.994%)	9442 (39.078%)	2423 (30.628%)	
*Hypertension*				<.00001
Yes	11,767 (36.695%)	7357 (30.451%)	4410 (55.773%)	
No	20,300 (63.305%)	16,803 (69.549%)	3497 (44.227%)	
*BMI*				<.00001
0–25	8941 (27.841%)	7371 (30.461%)	1570 (19.831%)	
25–30	10,729 (33.408%)	8346 (34.490%)	2383 (30.100%)	
>30	12,445 (38.751%)	8481 (35.048%)	3964 (50.069%)	
*Diabetes*				<.00001
Yes	4247 (13.234%)	2390 (9.884%)	1857 (23.477%)	
No	27,083 (84.394%)	21,318 (88.160%)	5765 (72.882%)	
Borderline	761 (2.371%)	473 (1.956%)	288 (3.641%)	
*Smoking*				<.00001
Never	17,537 (54.641%)	13,553 (56.041%)	3984 (50.360%)	
Quit smoking	7978 (24.857%)	5638 (23.313%)	2340 (29.579%)	
Some days	1208 (3.764%)	975 (4.032%)	233 (2.945%)	
Every day	5372 (16.738%)	4018 (16.614%)	1354 (17.115%)	
SIRI	1.250 ± 0.926	1.235 ± 0.821	1.419 ± 1.059	<.00001
NLR	2.188 ± 1.218	2.167 ± 1.086	2.382 ± 1.345	<.00001
AISI	313.372 ± 289.676	310.024 ± 244.172	358.615 ± 308.018	<.00001
PLR	126.048 ± 51.055	126.238 ± 47.311	130.384 ± 56.247	<.00001
MLR	0.282 ± 0.127	0.282 ± 0.116	0.302 ± 0.146	<.00001
SII	542.330 ± 376.259	537.854 ± 321.158	593.545 ± 384.118	<.00001

AISI = Aggregate Inflammatory Syndrome Index, BMI = body mass index, MLR = monocyte/lymphocyte ratio, NHANES = National Health and Nutrition Examination Survey, NLR = neutrophil/lymphocyte ratio, OAB = overactive bladder, PIR = poverty income ratio, PLR = platelet/lymphocyte ratio, SII = Systemic Inflammatory Index, SIRI = The Systemic Inflammatory Response Index.

* For categorical variables, *P* values were analyzed by Chi-square tests. For continuous variables, the *t* test for slope was used in generalized linear models.

Table 3 illustrates the connection between various systemic inflammatory indices (SIRI, NLR, AISI, PLR, MLR, and SII) and OAB. In Model 1, which did not adjust for covariates, we found that SIRI, NLR, AISI, PLR, MLR, and SII were positively correlated with OAB (SIRI: odds ratio [OR] = 1.219, 95% confidence interval [95% CI]: 1.187–1.252; NLR: OR = 1.150, 95% CI: 1.127–1.174; AISI: OR = 1.001, 95% CI: 1.000–1.001; PLR: OR = 1.001, 95% CI: 1.001–1.002; MLR: OR = 3.694, 95% CI: 3.056–4.466; SII: OR = 1.00, 95% CI: 1.000–1.000). These positive correlations persisted in both partially adjusted (Model 2) and fully adjusted models (Model 3). In sensitivity analyses, the systemic inflammatory indices were transformed from continuous variables into categorical variables using quartiles. In Model 3, participants in the highest quartile of SIRI, NLR, AISI, PLR, MLR, and SII had significantly higher OAB incidence compared to those in the lower quartiles, with an increase of 35% for SIRI, 35% for AISI, 6% for PLR, 27% for MLR, 39% for NLR, and 24% for SII (*P* for trend < .05).

**Table 3:** Association between SIRI, NLR, AISI, PLR, MLR, SII with OAB.

	Model1*			Model2†			Model3‡		
Characteristic	OR1	95% CI1	*P*-value	OR1	95% CI1	*P*-value	OR1	95% CI1	*P*-value
SIRI continuous variable	1.219	1.187–1.252	<.00001	1.230	1.195–1.267	<.00001	1.184	1.150–1.220	<.00001
Q1	Reference			Reference			Reference		
Q2	0.95	0.89–1.03	.2137	1.01	0.93–1.10	.7648	0.98	0.90–1.07	.646
Q3	1.13	1.05–1.21	.0015	1.21	1.11–1.31	<.0001	1.1	1.01–1.19	.0258
Q4	1.47	1.37–1.58	<.0001	1.55	1.43–1.67	<.0001	1.35	1.25–1.47	<.0001
*P* for trend	<.001			<.001			<.001		
NLR continuous variable	1.150	1.127–1.174	<.00001	1.133	1.108–1.158	<.00001	1.116	1.091–1.141	<.00001
Q1	Reference			Reference			Reference		
Q2	0.97	0.90–1.05	.4574	1.03	0.95–1.12	.4653	1.01	0.93–1.10	.724
Q3	1.08	1.00–1.16	.0495	1.13	1.04–1.22	.0027	1.09	1.00–1.18	.0499
Q4	1.47	1.37–1.57	<.0001	1.48	1.36–1.60	<.0001	1.39	1.28–1.50	<.0001
*P* for trend	<.001			<.001			<.001		
AISI continuous variable	1.001	1.000–1.001	<.00001	1.001	1.001–1.001	<.00001	1.000	1.000–1.001	<.00001
Q1	Reference			Reference			Reference		
Q2	0.96	0.89–1.03	.2354	0.98	0.91–1.07	.6889	0.94	0.87–1.02	.1514
Q3	1.09	1.01–1.17	.0205	1.16	1.07–1.25	.0003	1.06	0.98–1.15	.1538
Q4	1.38	1.28–1.48	<.0001	1.47	1.36–1.58	<.0001	1.28	1.18–1.38	<.0001
*P* for trend	<.001			<.001			<.001		
PLR continuous variable	1.001	1.001–1.002	<.00001	1.000	1.000–1.001	.00019	1.001	1.000–1.002	.00021
Q1	Reference			Reference			Reference		
Q2	0.87	0.81–0.94	.0002	0.87	0.80–0.94	.0004	0.93	0.86–1.00	.0635
Q3	0.91	0.85–0.98	.0089	0.86	0.80–0.93	.0002	0.97	0.90–1.05	.504
Q4	1.07	1.00–1.15	.0576	0.92	0.86–0.99	.0366	1.06	0.98–1.15	.1485
*P* for trend	<.001			<.001			<.001		
MLR continuous variable	3.694	3.056–4.466	<.00001	2.281	1.846–2.820	<.00001	2.632	2.116–3.275	<.00001
Q1	Reference			Reference			Reference		
Q2	0.99	0.92–1.07	.7991	1.01	0.93–1.09	.8891	1.05	0.97–1.14	.1979
Q3	1.05	0.97–1.13	.2397	1.04	0.96–1.13	.3098	1.1	1.01–1.20	.0275
Q4	1.35	1.25–1.45	<.0001	1.19	1.09–1.29	<.0001	1.27	1.16–1.38	<.0001
*P* for trend	<.001			<.001			<.001		
SII continuous variable	1.000	1.000–1.000	<.00001	1.000	1.000–1.000	<.00001	1.000	1.000–1.000	<.00001
Q1	Reference			Reference			Reference		
Q2	0.9	0.83–0.96	.0034	0.94	0.87–1.01	.105	0.91	0.84–0.98	.0186
Q3	1.06	0.98–1.14	.139	1.1	1.01–1.18	.0226	1.05	0.97–1.13	.2716
Q4	1.29	1.20–1.39	<.0001	1.36	1.26–1.47	<.0001	1.24	1.15–1.34	<.0001
*P* for trend	<.001			<.001			<.001		

95% CI = 95% confidence interval, AISI = Aggregate Inflammatory Syndrome Index, BMI = body mass index, MLR = monocyte/lymphocyte ratio, NLR = neutrophil/lymphocyte ratio, OAB = overactive bladder, OR = odds ratio, PLR = platelet/lymphocyte ratio, SII = Systemic Inflammatory Index, SIRI = the Systemic Inflammatory Response Index.

* Model 1, no covariates were adjusted.

† Model 2, age, sex, and race were adjusted.

‡ Model 3, age, sex, race, marital status, income to poverty ratio, education level, smoking status, BMI, hypertension, and diabetes were adjusted.

**Figure 2.**
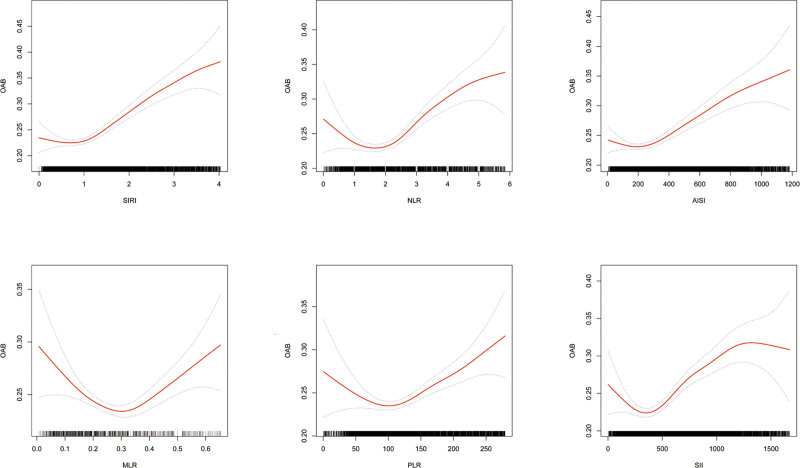
Smooth curve fitting for SIRI, NLR, AISI, PLR, MLR, SII with OAB. AISI = Aggregate Inflammatory Syndrome Index, MLR = monocyte/lymphocyte ratio, NLR = neutrophil/lymphocyte ratio, OAB = overactive bladder, PLR = platelet/lymphocyte ratio, SII = Systemic Inflammatory Index, SIRI = the Systemic Inflammatory Response Index.

Smoothed curve fitting was carried out to analyze the connection between OAB and the inflammatory indices SIRI, NLR, AISI, PLR, MLR, and SII. As shown in Figure 2 and Table 4, the breakpoints (K) for SIRI, NLR, AISI, PLR, MLR, and SII were 2.798, 4.182, 751.235, 96.389, 0.175, and 1114.75, respectively, with all log-likelihood ratio test *P*-values < .05. The smoothed curve fitting indicated that the connection between SIRI, NLR, AISI, PLR, MLR, SII, and OAB is nonlinear.

**Table 4:** Threshold effect analysis of SIRI, NLR, AISI, PLR, MLR, SII on OAB using a 2-piecewise linear regression model in Model 3.

	SIRI	NLR	AISI	PLR	MLR	SII
Standard linear model						
OR (95% CI)	1.184 (1.150–1.220)	1.116 (1.091–1.141)	1.000 (1.000–1.001)	1.001 (1.000–1.002) 0.0002	2.704 (2.036–3.592)	1.000 (1.000–1.000)
*P*-value	<.0001	<.0001	<.0001	.0002	<.0001	<.0001
Fitting by 2-piecewise linear model						
Breakpoint (K)	2.789	4.182	751.235	96.389	0.175	1114.75
OR1 (<K)	1.260 (1.204–1.319) < 0.0001	1.175 (1.137–1.214) < 0.0001	1.001 (1.001–1.001) < 0.0001	0.996 (0.993–0.998) 0.0009	0.071 (0.010–0.509) 0.0085	1.000 (1.000–1.001) < 0.0001
OR2 (>K)	1.085 (1.027–1.147) 0.0038	1.034 (0.992–1.077) 0.1151	1.000 (1.000–1.000) 0.0775	1.002 (1.001–1.002) < 0.0001	3.459 (2.530–4.729) < 0.0001	1.000 (1.000–1.000) 0.1724
OR2/OR1	0.861 (0.793–0.935) 0.0003	0.880 (0.829–0.934) < 0.0001	0.999 (0.999–1.000) < 0.0001	1.006 (1.003–1.009) < 0.0001	48.893 (6.054–394.880) 0.0003	1.000 (0.999–1.000) < 0.0001
Logarithmic likelihood ratio test *P*-value	<.001	<.001	<.001	<.001	<.001	<.001

Adjusted for age, race, education level, marital, PIR, diabetes, hypertension, smoking status, diabetes, and BMI.

95% CI = 95% confidence interval, AISI = Aggregate Inflammatory Syndrome Index, MLR = monocyte/lymphocyte ratio, NLR = neutrophil/lymphocyte ratio, OAB = overactive bladder, OR = odds ratio, PIR = poverty income ratio, PLR = platelet/lymphocyte ratio, SII = Systemic Inflammatory Index, SIRI = the Systemic Inflammatory Response Index.

**Figure 3.**
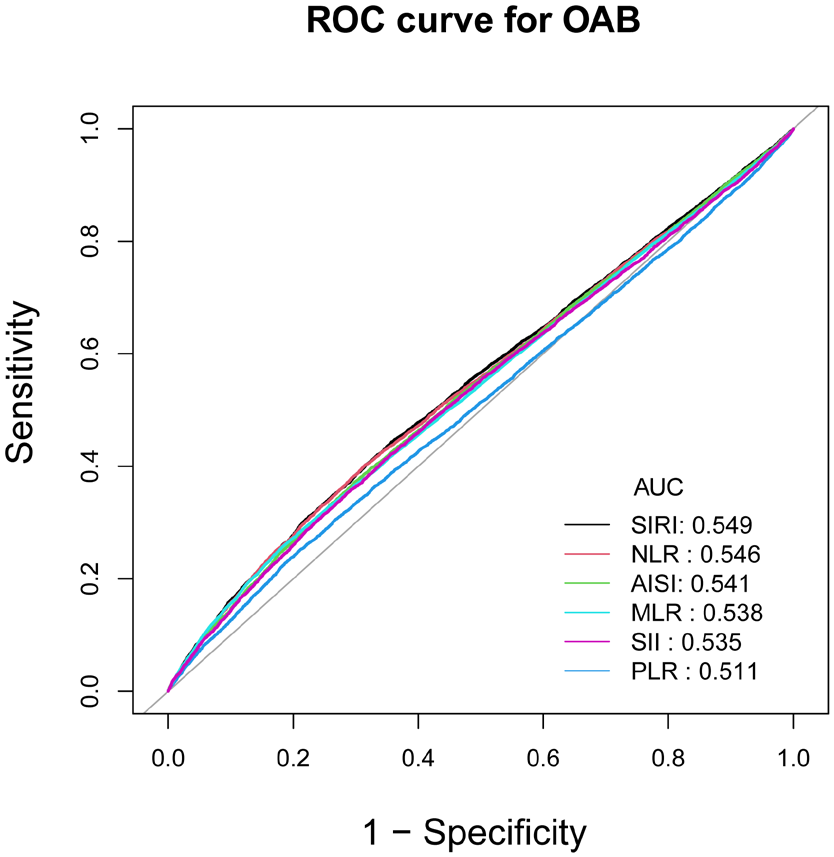
ROC curves and the AUC values of the 6 inflammatory markers (SIRI, NLR, AISI, PLR, MLR, and SII) in diagnosing OAB. AISI = Aggregate Inflammatory Syndrome Index, AUC = area under the curve, MLR = monocyte/lymphocyte ratio, NLR = neutrophil/lymphocyte ratio, OAB = overactive bladder, PLR = platelet/lymphocyte ratio, ROC = receiver operating characteristic, SII = Systemic Inflammatory Index, SIRI = the Systemic Inflammatory Response Index.

In this study, the ability of 6 inflammatory markers to identify patients with OAB was assessed using receiver operating characteristic (ROC) curves. ROC curve analysis showed that SIRI had significantly higher diagnostic efficacy for OAB compared to NLR, AISI, MLR, SII, and PLR. The areas under the curves for SIRI, NLR, AISI, MLR, SII, and PLR were 0.549, 0.546, 0.541, 0.538, 0.535, and 0.511, respectively.These findings suggest that SIRI may have higher discriminatory ability and accuracy than other anthropometric indices (NLR, AISI, MLR, SII, and PLR) in predicting the risk of OAB (Fig. 3 and Table 5).

**Table 5:** Comparison of AUC values for 6 inflammatory markers (SIRI, NLR, AISI, PLR, MLR, SII).

Test	AUC	95% CI low	95% CI upp	Best threshold	Specificity	Sensitivity	*P* for different in AUC
SIRI	0.549	0.542	0.557	1.306	0.683	0.404	Reference
NLR	0.546	0.534	0.549	2.329	0.691	0.39	.054
AISI	0.541	0.529	0.544	337.077	0.709	0.359	<.001
PLR	0.511	0.499	0.515	156.283	0.814	0.223	.078
MLR	0.538	0.525	0.539	0.354	0.828	0.235	.004
SII	0.535	0.523	0.538	560.023	0.659	0.401	<.001

95% CI = 95% confident interval, AUC = area under the curve, AISI = Aggregate Inflammatory Syndrome Index, MLR = monocyte/lymphocyte ratio, NLR = neutrophil/lymphocyte ratio, PLR = platelet/lymphocyte ratio, SII = Systemic Inflammatory Index, SIRI = the Systemic Inflammatory Response Index.

## 4. Discussion

In this cross-sectional investigation involving 32,115 U.S. adults, we found that several systemic inflammation markers, including SIRI, NLR, AISI, PLR, MLR, and SII, were positively connected to OAB. This positive correlation persisted even after adjusting for age, sex, race, degree of education, marital status, poverty income ratio, hypertension, diabetes, smoking status, and BMI, demonstrating that the association between these inflammation markers and OAB remained stable across various demographic groups. Additionally, the prevalence of OAB increased across the quartiles of each inflammation marker.

Numerous subsequent investigations have also looked into the connection between inflammation and OAB, although the underlying mechanisms remain unclear.^[18]^ It has been reported that chronic inflammation can cause functional changes in the bladder, leading to increased bladder sensitivity. In a controlled study, Koch et al^[19]^ found that chemokines and pro-inflammatory cytokines, such as MIP-1β, and SAA, were found to be considerably higher in OAB patients’ serum than in non-OAB patients. Our study delves deeper than previous research by not only examining the connection between OAB and SII, but also evaluating the predictive efficacy of SII levels on OAB incidence through ROC curve analysis. The emergence of systemic inflammatory response leads to the infiltration of cytokines, oxidative stress and immune cells in the body, and these changes will further damage the nerve endings innervating the bladder by destroying the urinary epithelial barriers, leading to an increase in the permeability of the vesicoureteral epithelium and bladder sensitivity, in addition to inflammatory response also causes infectious changes in cytokine receptors on the surface of the vesicoureteral epithelium, which induces a bladder mucosal sensitivity upregulation.^[12,13]^ When inflammation occurs in the body, the percentage of neutrophils increases, activating the corresponding cytokines and inducing apoptosis in lymphocytes, and these changes further induce oxidative stress, releasing reactive oxygen species and peptides, which ultimately cause chemokines, such as GRO-α and MIP-1β, to penetrate into the tissue of the bladder wall from the urine and disrupt the endothelial integrity of the bladder, and these findings are more compelling evidence for the correlation between the inflammation response correlates with OAB.^[16]^ Additionally, we analyzed the correlation between SIRI, NLR, AISI, PLR, MLR, and OAB, offering a more comprehensive analysis than previous studies.

Systemic inflammation acts through immune-mediated pathways to regulate bladder function. Chronic inflammation is associated with detrusor overactivity, a key feature of OAB. Elevated levels of inflammatory markers, including neutrophils and platelets, lead to oxidative stress, cytokine release, and bladder uroepithelial dysfunction. These processes may lead to increased bladder hypersensitivity and altered afferent nerve signaling, which are critical to the pathogenesis of OAB.^[20,21]^ Platelet activation and neutrophil infiltration have been shown to exacerbate local inflammatory responses, leading to increased production of pro-inflammatory cytokines such as interleukin-6 and tumor necrosis factor-alpha. These cytokines may disrupt normal bladder signaling, promoting urgency and frequency symptoms characteristic of OAB.^[13]^ An increasing number of studies are exploring the potential diagnostic role of multiple systemic inflammatory biomarkers in various diseases, with SIRI, NLR, AISI, PLR, MLR, and SII being the most frequently mentioned. SIRI, computed as neutrophils × monocytes/lymphocytes, serves as a reliable indicator of the inflammatory response due to the roles of neutrophils in natural immunity, monocytes in immune defense,^[22,23]^ and lymphocytes in immune system regulation through cytokine secretion. According to a study by Kim et al, the NLR was considerably greater in OAB-positive women than in non-OAB-positive women, and NLR levels were strongly associated with the intensity of symptoms of OAB.^[24]^ The systemic AISI, is another indicator of systemic inflammatory status based on a combination of whole blood cell counts. AISI is recognized as a novel prognostic biomarker, and while PLR and MLR are also used in disease prediction.^[25,26]^ Zinellu et al^[27]^ found that AISI had a higher predictive value for inflammatory disease prognosis than MLR and PLR. The SII, is widely studied due to the role of platelets in immune-inflammatory responses.^[16]^ SII effectively represents systemic inflammation and has been shown to correlate with the severity of OAB.^[28]^

Earlier studies have primarily focused on single inflammatory markers in relation to OAB,^[29,30]^ but there are still very few studies that explore the relationship between multiple systemic inflammatory response markers and OAB. Our study offers several advantages in this context. First, the correlation between SIRI, NLR, AISI, PLR, MLR, and SII and OAB has been less frequently investigated, with existing studies showing some discrepancies in their results. Our research provides new insights into the connection between OAB and certain systemic inflammatory indicators in the U.S. population and examines the correlations among these markers. Second, we assessed the connection between the levels of SIRI, NLR, AISI, PLR, MLR, and SII and the incidence of OAB using the quartile method. Our findings suggest these 6 indicators of systemic inflammation as potential biomarkers for identifying individuals at higher risk of developing OAB. Moreover, these markers are easily obtained from blood tests, practical and cost-effective, and these indicators provide new perspectives for future disease diagnosis.

Our research has a number of other drawbacks. Firstly, because we collected data from NHANES and employed a study design that is cross-sectional, we were not able to prove causation relationships between the multiple systemic inflammatory markers and OAB. The serologic markers collected were representative of a single examination, which limits the ability to assess real-time inflammatory markers and the overall immune status of the patients. Additionally, although we adjusted for multiple covariates that we believed might influence the results, the potential impact of some unmeasured factors cannot be entirely ruled out. Therefore, larger sample numbers and prospective research are required in the future to further validate these findings.

## 5. Conclusion

OAB is a disease that severely affects the quality of life and socialization of patients. Current studies have shown that elevated levels of SIRI, NLR, AISI, PLR, MLR, and SII are strongly associated with the likelihood of developing OAB, and that the higher the levels of these inflammatory markers, the greater the risk of developing OAB. This suggests that SIRI, NLR, AISI, PLR, MLR, and SII may be important indicators for the diagnosis of OAB. Given the association between systemic inflammation and OAB, further prospective studies could be conducted to evaluate whether anti-inflammatory therapies could be integrated into OAB management strategies to improve patient prognosis.

## Acknowledgments

We extend our gratitude to the NHANES databases for providing access to this valuable data.

## Author contributions

**Conceptualization:** Mingchu Jin, Heng Liu.

**Data curation:** Mingchu Jin, Jie Xu, Haidong Hao, Yutang Yuan.

**Methodology:** Mingchu Jin.

**Resources:** Mingchu Jin.

**Software:** Mingchu Jin, Heng Liu, Jie Xu, Haidong Hao.

**Supervision:** Yu Zhou.

**Validation:** Mingchu Jin, Yu Zhou.

**Writing – original draft:** Mingchu Jin, Jie Xu.

**Writing – review & editing:** Mingchu Jin, Hongtao Jia.
